# Estimating background rates of Guillain-Barré Syndrome in Ontario in order to respond to safety concerns during pandemic H1N1/09 immunization campaign

**DOI:** 10.1186/1471-2458-11-329

**Published:** 2011-05-17

**Authors:** Shelley L Deeks, Gillian H Lim, Mary Anne Simpson, Laura Rosella, Christopher O Mackie, Camille Achonu, Natasha S Crowcroft

**Affiliations:** 1 Ontario Agency for Health Protection and Promotion, Toronto, Ontario, Canada; 2 Dalla Lana School of Public Health, University of Toronto, Toronto, Ontario, Canada; 3 Ontario Ministry of Health and Long-Term Care, Toronto, Ontario, Canada; 4 City of Hamilton Public Health Services, Hamilton, Ontario, Canada; 5 Department of Clinical Epidemiology and Biostatistics, McMaster University, Hamilton, Ontario, Canada; 6 Department of Laboratory Medicine and Pathobiology, University of Toronto, Ontario, Canada

## Abstract

**Background:**

The province of Ontario, Canada initiated mass immunization clinics with adjuvanted pandemic H1N1 influenza vaccine in October 2009. Due to the scale of the campaign, temporal associations with Guillain-Barré syndrome (GBS) and vaccination were expected. The objectives of this analysis were to estimate the number of background GBS cases expected to occur in the projected vaccinated population and to estimate the number of additional GBS cases which would be expected if an association with vaccination existed. The number of influenza-associated GBS cases was also determined.

**Methods:**

Baseline incidence rates of GBS were determined from published Canadian studies and applied to projected vaccine coverage data to estimate the expected number of GBS cases in the vaccinated population. Assuming an association with vaccine existed, the number of additional cases of GBS expected was determined by applying the rates observed during the 1976 Swine Flu and 1992/1994 seasonal influenza campaigns in the United States. The number of influenza-associated GBS cases expected to occur during the vaccination campaign was determined based on risk estimates of GBS after influenza infection and provincial influenza infection rates using a combination of laboratory-confirmed cases and data from a seroprevalence study.

**Results:**

The overall provincial vaccine coverage was estimated to be between 32% and 38%. Assuming 38% coverage, between 6 and 13 background cases of GBS were expected within this projected vaccinated cohort (assuming 32% coverage yielded between 5-11 background cases). An additional 6 or 42 cases would be expected if an association between GBS and influenza vaccine was observed (assuming 32% coverage yielded 5 or 35 additional cases); while up to 31 influenza-associated GBS cases could be expected to occur. In comparison, during the same period, only 7 cases of GBS were reported among vaccinated persons.

**Conclusions:**

Our analyses do not suggest an increased number of GBS cases due to the vaccine. Awareness of expected rates of GBS is crucial when assessing adverse events following influenza immunization. Furthermore, since individuals with influenza infection are also at risk of developing GBS, they must be considered in such analyses, particularly if the vaccine campaign and disease are occurring concurrently.

## Background

After the emergence of pandemic influenza in April 2009, a safe and effective vaccine needed to be developed and approved under significant time constraints. Arepanrix™ H1N1 vaccine, an adjuvanted influenza vaccine, was authorized for sale in Canada on October 13, 2009 [1]. It was anticipated that this vaccine would have a similar safety profile as seasonal influenza vaccines; however, it contained an adjuvant that had not been used in population-based settings in North America prior to the pandemic. As a result there was suggestion in the popular press of public mistrust in the vaccine [2,3]. In addition, it was recognized that due to the scale of the campaign, being the largest immunization campaign in Canadian history, a number of events would be temporally associated with the vaccine regardless of any causal associations. It was expected that these events would trigger intense media interest, as well as require public health attention and resources.

Adverse event following immunization (AEFI) surveillance is a critical component of any immunization program, especially a campaign targeting the entire population using a new vaccine with a novel adjuvant. Safety signals are expected to occur and must be assessed thoroughly. This is important for program integrity, as spurious associations could undermine public confidence. Adverse events need to be assessed in order to determine if any causal, rather than temporal, association exists; however, these assessments are often time-consuming and causality is difficult to infer. This is especially true for Guillain-Barré syndrome (GBS), a relatively rare condition that was causally-linked to the 1976 swine-origin influenza A (H1N1) vaccination campaign in the United States (US) [4].

GBS is the leading cause of acute flaccid paralysis in developed countries and is characterized by various degrees of weakness, sensory abnormalities and autonomic dysfunction [5]. The estimated annual incidence in Canada is between 1.0 and 2.3 per 100,000 population [6,7]. GBS can occur at any age, but incidence increases with age, and is more common among males than females [6]. The etiology of GBS is not completely understood, however a number of gastrointestinal and respiratory infectious triggers have been identified, with *Campylobacter *being the most common. The risk after *Campylobacter jejuni *has been estimated to be 1 case of GBS per 1000 *C. jejuni *cases [8]. This can be compared with a risk after influenza infection approximately two orders of magnitude lower at 4-7 GBS cases per 100,000 cases of influenza. GBS has also been associated with vaccines. A recent review of studies examining associations between various vaccines and GBS found that, with rare exceptions, these associations have been only temporal, with little evidence with most vaccines to support a causal association [5]. The evidence for a causal association is strongest for the swine influenza vaccine that was used in the US in 1976-77; the US immunization program was suspended in 1976 following increased reports of GBS after vaccine administration. Relative risks of between 4 and 8 for the 6 to 8 week post-vaccination period were demonstrated [4]. In contrast, studies of associations between seasonal influenza vaccines have found only small or no increased risk of GBS. Lasky and co-authors estimated an increased risk of GBS after seasonal influenza vaccination of 1 per 1,000,000 vaccinated persons [9]. Comparing the risk estimates of GBS after influenza infection to that after influenza vaccination reveals that the risk of GBS following infection is 40 - 70 times greater than the risk, if it exists, of GBS following seasonal influenza vaccine [8,10].

The objectives of the analysis were to estimate the number of background GBS cases expected to occur in the projected vaccinated population in the province of Ontario during the 2009 pandemic influenza immunization campaign and to estimate the number of additional GBS cases which would be expected if previous associations between GBS and influenza vaccines were seen. For comparison purposes, we also estimated the number of GBS cases that would be expected to occur among persons infected with pandemic influenza during the immunization campaign.

## Methods

**Figure 1:**
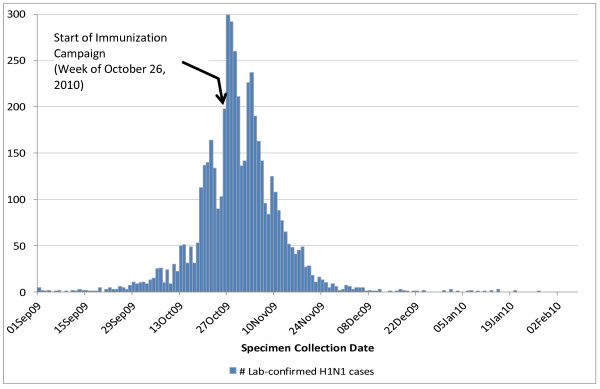
**Epidemic curve of laboratory-confirmed H1N1 cases in Ontario during the 2nd wave (N = 5,029)**.

We conducted an ecologic analysis in the province of Ontario during the second wave of the influenza pandemic (Figure 1).

### Background cases of GBS among vaccinated cohort

Expected background rates of GBS were determined from two Canadian sources and applied to the Ontario population (12.8 million) using a method similar to that described by Black and co-authors [11]. Juurlink and co-authors reported a rate of 1.4 per 100,000 in Ontario and Hauck and co-authors reported age-sex adjusted GBS rates over an 11 year period of between 0.97 to 2.32 per 100,000 per year in Alberta [6,7]. The Alberta range was used for calculating the background rates as the Ontario point estimate was included within. The rates include GBS cases from all causes. The expected number of GBS cases applies to the entire population unless otherwise stated. Ontario demographic data were obtained from Statistics Canada [12].

GBS is not a reportable condition in Ontario. Therefore in order to determine the number of expected temporally-reported GBS cases among vaccinated persons, we estimated the number of H1N1 vaccine doses administered in the province (i.e., vaccine coverage) and then applied the background GBS rates to estimate the number of expected GBS cases that would occur in the vaccinated cohort over time. Local public health units in the province delivered the vast majority of doses of vaccine through H1N1 vaccination clinics held between approximately October 26 and December 27, 2009; however, vaccine was also delivered through other delivery agents including physicians and through community influenza clinics offered by community health centres, community care access centres, hospitals, long-term care homes, as well as pharmacy-based clinics and workplace clinics. The end date of the clinics varied by health unit and tended to be demand driven and some vaccine delivery continued beyond December 2009.

**Figure 2:**
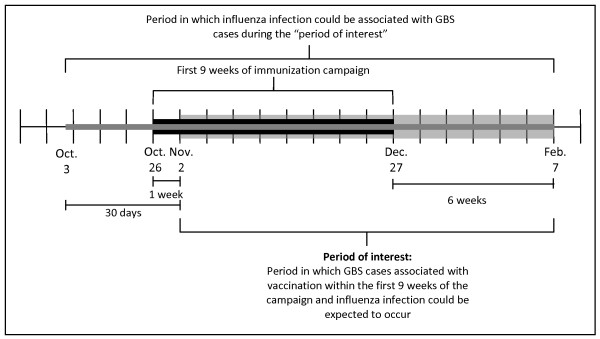
**Relevant timelines of interest in GBS analysis**.

The projected distribution of the vaccinated population within the first nine weeks of the campaign (October 26 to December 27, 2009) was based on publicly available data [13-15]. The overall provincial coverage estimate during the second wave of the pandemic was assumed to be 38%, based on publications from the Ontario Ministry of Health and Long-Term Care [14,15]. For comparison, as a form of sensitivity analysis, 32% coverage was also considered [16,17]. Next, the distribution of the weekly number of doses administered was based on the weekly number of doses distributed to Ontario, as reported by the Public Health Agency of Canada [13]. Keeping the cumulative coverage fixed at 32% or 38%, the weekly number of doses distributed was then multiplied by a series of parameter estimates believed to reflect the variability in public demand, capacity and contributions of vaccine delivery agents across the province, to derive the projected weekly distribution of the vaccinated cohort. To determine the expected number of GBS cases occurring among vaccinated persons, the expected number of cases in the population was multiplied by the estimated vaccine coverage. We assumed that the daily/weekly rate of developing GBS was constant. A vaccinated person was considered as eligible for having an incident diagnosis of GBS from 1 to 6 weeks following vaccination as this is typically the risk period considered for GBS and vaccination [4,7,9]. GBS cases occurring during the same week as vaccination would not be associated with vaccination. As a weekly rate was used, a vaccinated cohort would be eligible to be diagnosed with GBS in each of the six weeks after vaccination; after which any GBS case would be considered independent of vaccination and thus were no longer eligible to contribute to the burden. Therefore, the period of interest for this investigation (i.e., the period in which GBS cases associated with vaccination could be expected to occur), was between November 2, 2009 and February 7, 2010. This is illustrated in Figure 2.

### Estimated number of additional GBS cases among vaccinated cohort

The number of additional cases of GBS expected among the vaccinated cohort if an association was seen which was similar to either that experienced during the 1976 vaccination campaign or during the 1992/1993 and 1993/1994 seasonal campaigns was determined by applying the rates identified by Schonberger and co-authors, and Lasky and co-authors, respectively, to the vaccinated population [4,9]. The following formula was used to estimate the total number of additional GBS cases that might be expected to occur among individuals who were vaccinated during the first nine weeks of the campaign:

where

• i denotes the week in which the projected cohort of individuals was vaccinated

• j denotes the number of weeks relative to the start of the immunization campaign

• cohort_i_: projected number of individuals vaccinated in week i

• risk_j-i _= risk of GBS j-i weeks after vaccination

For comparison, the number of GBS cases reported to the province through the integrated Public Health Information System (iPHIS) by local public health units as part of their passive surveillance of AEFI during the period of interest (as defined above) was obtained from the Ontario Ministry of Health and Long-Term Care (MOHLTC). Reported cases of GBS were assessed by the MOHLTC using the Brighton Criteria based on a review of consultation notes of the attending neurologist and laboratory results.

### Estimated number of GBS cases among persons infections with influenza

The number of GBS cases expected to occur as a result of influenza infection was determined based on risk estimates of GBS after influenza-like illness (ILI) identified by Stowe and co-authors [10]. These rates were applied to estimates of the cumulative incidence of influenza infection in the population of Ontario using two different sources of data. The first estimate was the number of laboratory confirmed influenza cases occurring in Ontario between October 3, 2009 and February 7, 2010. This time period was selected as GBS cases have occurred between 0 and 30 days after ILI [10]. Therefore influenza cases occurring between the aforementioned dates could result in GBS cases being reported during the period of interest (November 2, 2009 and February 7, 2010). This is also illustrated in Figure 2. Laboratory-confirmed cases were determined from the public health laboratory-based information system Labware. As testing for H1N1 also occurred within private laboratories and hospitals, the data from Labware represented approximately 70% of all laboratory-confirmed cases in Ontario during the second wave of the pandemic, relative to data captured within the provincial surveillance information system iPHIS (unpublished data). However, access to laboratory testing was limited during the second wave of the pandemic and would underestimate both population infection and influenza-associated GBS cases. Furthermore, laboratory-confirmed cases will exclude those who were infected but did not get tested for their illness. Therefore we also used data from a seroprevalence study conducted in Ontario to gain a more comprehensive estimate of the number of cases of influenza occurring in the province that includes a broader spectrum of illness that is also not sensitive to laboratory testing changes. The seroprevalence study, which was based on hemagglutination inhibition assay testing, revealed that 13.7% of Ontarian adults who did not report receiving H1N1 vaccine were infected during the second wave [18]. We applied this proportion to the estimated unvaccinated Ontario population to determine the total number of new influenza cases occurring between October 3, 2009 and February 7, 2010. We then applied the distribution of laboratory-confirmed cases to this total value to derive the daily number of new influenza cases during the same period. Lastly, using the relative risk estimate (16.64) for GBS in the 0 to 30 days following ILI, as reported by Stowe and co-authors [10], we assumed that the daily risk during this 31-day period was constant and applied this to the daily number of influenza cases in order to derive daily estimates of GBS cases associated with influenza infection.

## Results

The distribution of laboratory-confirmed influenza cases in Ontario during the second wave of the pandemic is provided in Figure 1. During the 9 weeks of Ontario's H1N1 vaccination campaign (i.e., between October 26-December 27, 2009), between 22 and 52 GBS cases were expected to occur in the province in its entirety, based on the background rates of all-cause GBS. We projected that by December 27, 2009, between 4.2 and 5 million Ontarians (between 32%-38% of the population), were vaccinated. Table 1 reveals the estimated baseline number of GBS cases occurring among vaccinated individuals by week of the campaign, starting one week after campaign commencement and extending 6 weeks beyond campaign conclusion. Assuming 38% coverage, we estimated that between 6 and 13 coincidental cases of GBS should have occurred among vaccinated persons during the period of interest (November 2, 2009 to February 7, 2010). By comparison, between 5 and 11 coincidental GBS cases should have occurred if 32% coverage was assumed. During the same time period, 7 cases of GBS were reported among vaccinated persons in the province.

**Table 1:** Baseline number of GBS cases among vaccinated individuals, November 2, 2009 - February 7, 2010

Week of Vaccination Campaign	Projected number of individuals vaccinated in Ontario		Projected cumulative baseline number of GBS cases	
	
	32% coverage	38% coverage	32% coverage	38% coverage
Week 1: Oct 26 - Nov 1	240,322	285,382	N/A	N/A

Week 2: Nov 2 - Nov 8	418,444	496,902	0.04 - 0.11	0.05 - 0.13

Week 3: Nov 9 - 15	519,186	616,533	0.17 - 0.40	0.20 - 0.48

Week 4: Nov 16 - 22	970,861	1,152,898	0.39 - 0.93	0.46 - 1.1

Week 5: Nov 23 - 29	1,057,184	1,255,406	0.79 - 1.9	0.94 - 2.2

Week 6: Nov 30 - Dec 6	634,192	753,103	1.4 - 3.3	1.6 - 3.9

Week 7: Dec 7 - 13	239,874	284,850	2.1 - 5.0	2.5 - 6.0

Week 8: Dec 14 - 20	72,191	85,726	2.8 - 6.7	3.3 - 8.0

Week 9: Dec 21 - 27	29,887	35,491	3.5 - 8.3	4.1 - 9.9

Week 10: Dec 28 - Jan 3	0	0	4.0 - 9.6	4.8 - 11.4

Week 11: Jan 4 - 10	0	0	4.4 - 10.5	5.2 - 12.5

Week 12: Jan 11 - 17	0	0	4.6 - 11.0	5.5 - 13.0

Week 13: Jan 18 - 24	0	0	4.7 - 11.1	5.5 - 13.2

Week 14: Jan 25 - 31	0	0	4.7 - 11.2	5.6 - 13.3

Week 15: Feb 1 - 7	0	0	4.7 - 11.2	5.6 - 13.3

Assuming 38% coverage, if an association between GBS and influenza vaccine similar to that seen in 1976 was observed, we would expect to see an additional 42 cases of GBS over baseline among the vaccinated population (Table 2), for a total of between 48 and 55 cases GBS cases. If, however, an association was seen that was similar to previous seasonal influenza vaccine, we would expect an additional 6 cases during the same time period, for a total of between 12 and 19 GBS cases among the vaccinated population. Estimates assuming 32% coverage were similar, though slightly lower (between 5 and 35 additional cases).

**Table 2:** Additional number of GBS cases among vaccinated individuals, November 2, 2009 - February 7, 2010

	Projected cumulative additional number of GBS cases			
	
Week of Vaccination Campaign	1992/94 Seasonal Flu **(Lasky et al **[10]**)**		1976 Swine Flu **(Schonberger et al **[4]**)**	
	
	32% coverage	38% coverage	32% coverage	38% coverage
Week 1: Oct 26 - Nov 1	N/A	N/A	N/A	N/A

Week 2: Nov 2 - Nov 8	0.01	0.02	0.19	0.23

Week 3: Nov 9 - 15	0.16	0.19	1.2	1.4

Week 4: Nov 16 - 22	0.45	0.54	3.4	4.0

Week 5: Nov 23 - 29	0.89	1.1	7.1	8.4

Week 6: Nov 30 - Dec 6	1.6	1.9	12.6	15.0

Week 7: Dec 7 - 13	2.5	3.0	19.7	23.4

Week 8: Dec 14 - 20	3.3	3.9	26.2	31.1

Week 9: Dec 21 - 27	3.8	4.5	30.6	36.4

Week 10: Dec 28 - Jan 3	4.2	4.9	33.1	39.3

Week 11: Jan 4 - 10	4.4	5.2	34.3	40.8

Week 12: Jan 11 - 17	4.5	5.4	34.8	41.3

Week 13: Jan 18 - 24	4.6	5.4	34.9	41.5

Week 14: Jan 25 - 31	4.6	5.5	35.0	41.5

Week 15: Feb 1 - 7	4.6	5.5	35.0	41.5

There were 5,029 laboratory confirmed cases of influenza occurring in Ontario between September 1, 2009 and February 7, 2010, however only 4,921 (97.9%) occurred between October 3 2009 and February 7, 2010, the time period in which influenza infection could have been associated with GBS cases during the "period of interest". These latter influenza cases would have been associated with less than 1 (0.06-0.14) GBS case. Seroprevalence data revealed that 13.7% of unvaccinated individuals (N = 1,110,097 assuming 38% coverage) were newly infected with influenza during the second wave of the pandemic. This would have resulted in 1,087,862 influenza cases which would be associated with between 12.9 and 30.8 GBS cases during the period of interest. Therefore when taking both methods of estimating influenza illness into account, during the same time period examined for the vaccinated cohort, between 0.06 and 30.8 GBS cases could be expected to be associated with influenza infection within the unvaccinated population.

## Discussion

This study estimated the expected number of GBS cases during and after the H1N1 mass vaccination campaign in Ontario, Canada. We propose a method to estimate these cases based on vaccine coverage, number of influenza infections and the respective timing of each in the population.

In Ontario, 7 cases of GBS were reported among vaccinated persons during the period of interest. This was well within the expected background rate of GBS estimated in this study and does not suggest an increased number of GBS cases due to the vaccine. Elsewhere in Canada and internationally there have been similar conclusions. In an April 27, 2010 web posting, the Public Health Agency of Canada, indicated that 31 GBS cases had been reported following vaccination in Canada (approximately 1.2 cases per million doses distributed) and that concerns about GBS had not emerged in connection with H1N1 vaccines [19]. In Sweden, as of April 16, 2010 there had been 13 GBS reports, lower than would be expected considering the size of the vaccinated population [20]. Similarly, in the United Kingdom, 10 GBS cases were reported until March 16, 2010, and it was concluded that there was no evidence across Europe that H1N1 vaccines caused GBS [21]. In the US, Vellozzi and co-authors found that the GBS reporting rates among persons vaccinated with pandemic vaccine was less than the expected background GBS rates [22]. However, an earlier US publication by Prothro and co-authors reported preliminary results of surveillance for GBS after receipt of H1N1 vaccine which revealed an elevated, statistically significant association between 2009 H1N1 vaccination and GBS and an attributable risk of 0.8 excess cases of GBS per 1 million vaccinations; importantly, 59% of the vaccinees also reported antecedent respiratory illness symptoms in the 42 days before GBS onset [23]. Given that the risk of GBS following influenza is greater than the risk following seasonal vaccine, illness rather than vaccination may have been the causal factor.

In addition to the background risk of GBS and the potential risk introduced by vaccination, individuals with influenza infection are also at risk of developing GBS. Vaccinated individuals may have become infected with influenza prior to developing immunity to the vaccine and therefore would be at risk of developing influenza-associated GBS. These individuals pose a particular challenge if they develop GBS as causality is more difficult to elucidate. Not accounting for this risk in an analysis of associations between GBS and influenza vaccine could result in associations inaccurately being attributed to the vaccine rather than the disease, particularly when the risk of developing GBS is 40-70 times greater after influenza than after vaccine [8,10]. This is an especially important consideration during a pandemic [24], when it can be expected that influenza cases and vaccination is occurring concurrently (i.e., they are not mutually exclusive populations); indeed, a large burden of influenza infection in Ontario occurred prior to availability of vaccination (Figure 1). This is in contrast to seasonal influenza when the majority of vaccination occurs prior to the onset of the influenza season. In our analysis, up to 31 influenza-associated GBS cases could be expected to occur among individuals who were infected with influenza during the period of interest.

There are many limitations in this study. Coverage estimates were estimated as accurate and timely coverage data at the provincial level were not available in real-time during the campaign. While the coverage estimates used in this study are potentially subject to misclassification, they were based on the best information available at the time. Overestimating coverage would have resulted in a higher estimate of the number of baseline and additional number of GBS cases within the vaccinated cohort, while underestimating coverage would have resulted in lower estimates. A provincial report on the pandemic cited an estimate of coverage that agreed with our overall estimate of 38% [15], while estimates cited from other sources [14] including surveys [16,17] were within the range explored in this analysis. We were reassured by the results of our sensitivity analyses that a lower coverage estimate of 32% did not change our overall conclusions. Age-sex standardized GBS rates would overestimate the background number of GBS cases, as the vaccinated population is younger than the general population and GBS incidence increases with age. However, we calculated the Alberta age-standardized GBS rate among persons less than 60 years of age and the number of background GBS cases that we would expect would be 7 cases, still within the calculated range. Although health care providers are legally required under the Ontario Health Protection and Promotion Act to report serious adverse events following immunization, the number of GBS cases reported to the province during the period of interest may have been underestimated as it was based on a passive surveillance system that relied on providers' notification to the local public health unit. Providers may not have reported the GBS case if they did not believe it was related to the vaccine, or they may have reported it directly to the vaccine manufacturer, thus by passing the provincial reporting system. However, given the heightened awareness surrounding GBS and influenza immunization, it is unlikely that this occurred frequently. Further, the number of GBS cases in the province was still well below the upper limit of the expected coincidental GBS baseline range. Finally, when calculating the number of influenza-related cases of GBS occurring among the unvaccinated population, we used risk estimates of GBS after ILI rather than influenza infection. This may have resulted in either an over- or under-estimate of the number of influenza-associated GBS cases depending on whether influenza is less or more likely to be associated with GBS than ILI. We also made assumptions regarding the actual number of influenza cases that occurred which produced a wide range of estimated GBS cases among the unvaccinated population. Assuming that only the unvaccinated population remained at risk of influenza (and thereby influenza-associated GBS) would underestimate the number of cases, as infection with pandemic influenza occurred concurrently with vaccination. Therefore some vaccinated persons could also have been infected with influenza and thus eligible for influenza-associated GBS.

Despite these limitations, the approach used in this study to estimate expected GBS cases was extremely helpful during the H1N1 vaccination campaign to rapidly compare with real-time AEFI reports of GBS allowing us to detect a potential signal very quickly. Background rates were calculated in advance of the campaign, as suggested by Black and co-authors [11], therefore all data were available prior to receiving AEFI reports. The estimates were adjusted in real time as vaccine coverage data became available. During this process a GBS case occurred in a vaccine recipient [25]. The data were used to provide context to the situation. At that point in time (ie November 28, 2009) we would have expected between 1 and 2 background cases of GBS reported in the province among a vaccine recipient, and this was the first case that had been reported. Having timely information available greatly assisted with communications, allowed the media to provide a balanced perspective and helped maintain public confidence in the vaccination campaign.

During a pandemic, public health officials should ideally have access to timely comprehensive vaccination coverage data from an immunization registry and accurate data regarding GBS and other AEFI from disease registries and patient reports. There is no doubt that this would assist in assessing vaccine-associated adverse events. Unfortunately, this ideal does not exist in many jurisdictions and we are left with non-optimal methods for assessing these events. AEFI surveillance is a core function of public health that must proceed regardless of data limitations. Our methodology, which was relatively easy to employ, could be adapted by many jurisdictions to assist with both AEFI assessment and communications during mass immunization campaigns.

## Conclusions

Awareness of expected rates of GBS is crucial when assessing adverse events following influenza immunization, particularly when an entire population is eligible for vaccine. Although ecologic in nature, our analysis does not support an association between H1N1 vaccine and GBS as the number of cases reported was within the expected range of background cases occurring in the province. Attribution of causality for GBS is difficult; however, it is essential that the risk of GBS following influenza is taken into account during a pandemic when vaccination and disease are occurring concurrently to avoid making spurious associations.

## Competing interests

The authors declare that they have no competing interests.

## Authors' contributions

SD was the primary author of this paper and was responsible for the overall study design with contributions from GL, CM and NC. GL was responsible for the compilation, validation and analysis of data, with contributions from SD, CA, NC, and LR. MAS and CA provided some of the data used in this analysis. All authors contributed to the interpretation of the data, critically reviewed the manuscript and approved the final version of the manuscript.

## Pre-publication history

The pre-publication history for this paper can be accessed here:

http://www.biomedcentral.com/1471-2458/11/329/prepub
